# Dissertation upon a New Species of Emphysema, Developed after Abundant Hemorrhage

**Published:** 1832-12

**Authors:** 


### BIBLIOGRAPHICAL NOTICES.

*Dissertation upon a new Species of Emphysema, developed after abundant Hemorrhage.* By M. E. Rebolle de Gex.—*Gazette Médicale,* t. 3, No. 103.

The existence of gas in the blood-vessels has long been known: nothing is more common than to find, upon dissection of patients who have died of different diseases, but particularly of typhus gravior, a certain quantity of gas mingled with the blood in the veins, which appears to arise from decomposition of the blood. Such, however, is not the kind of emphysema described by M. Rebolle, which appears to arise only after abundant hemorrhages: the following case is an example of it.

*Profuse Epistaxis; Death; Gas in the whole of the Venous System.*

Ducret, æt. fifteen, was admitted into the Hôtel Dieu, with continued inflammatory fever, which was relieved by leeches, abstinence, and rest; but it was followed by an attack of quotidian fever, which quickly yielded to the exhibition of salicine. The patient was still occasionally subject to epistaxis, with which he had been attacked previous to the occurrence of the fever; and, at the time he was about to quit the hospital, he was found in the night bathed in his blood. The quantity of blood he had lost was very great. The nostrils were immediately plugged, and the patient was thus saved from immediate death.

On the following day, he was in a state of great exhaustion: the eyes were dull, his strength gone, and the pulse at the wrist thready and wavering; it was evident that he could not long survive. In the course of the day, general œdema began to be manifested: he became weaker and weaker, and died on the third day.

*Dissection, fifteen hours after death.* The body presented no appearance of putrefaction. Almost every part was œdematous; the lungs most so, and next the cellular tissue. In the right auricle and ventricle of the heart, a clot of feeble consistence was found, and of a light colour: this clot was emphysematous; it contained numerous small cells. A smaller clot, of the same kind, was found in the left auricle. The vena cava, subclavian, axillary, and internal jugular veins, as well as the iliac and femoral, contained, in different points of their course, many small bubbles of air, which were separated from each other by small drops of blood, the colour of which was less black than natural. This appearance was more marked in the smaller veins: it resembled a spirit-of-wine thermometer, into which air has been inserted bubble after bubble. Upon dividing the vessels, gas escaped mixed with blood. No air could be detected in the arteries.

Another case of a similar kind, and experiments upon animals, lead to the same conclusion, that gas is present in the circulating system after profuse hemorrhages. But what is the nature of this gas? Is it atmospheric air introduced by absorption, or is it the result of spontaneous formation, consecutive to hemorrhage? This question might have been solved by chemical experiments; but M. Rebolle, probably, could not collect a sufficient quantity of the gas for that purpose. He shews, by the following experiment, that the gas could not be atmospheric air absorbed by the vessels from which the bleeding proceeded. After having exposed the two crural veins of a young dog, he plunged him up to the neck into a bath at twenty degrees; he then divided the crural veins, and the crural artery, without removing the creature from the water. After death, the heart and venous system contained gas, as in other cases. Still this experiment does not shew, as M. R. imagines it does, that the presence of the air must be attributed to pulmonary absorption: he should first have proved the existence of atmospheric air, or its element, in the blood-vessels. But whatever may be the cause to which we attribute the presence of air in the blood-vessels, the following case cannot be uninteresting.

*Schirrous Tumor on the Back; Removal of it followed by considerable Hemorrhage; Emphysema; Death. The Emphysema depending on the Presence of inflammable Gas.*

Duclos, æt. twenty-five, of a strong constitution, had a very large tumor removed from his back, on the 25th of February: the tumor was very extensive, and the operation lasted forty-five minutes. There was great difficulty in securing the divided vessels, and the hemorrhage was profuse. For a long time the patient remained in a state of alarming syncope.

26th. Rather better; belly supple, retracted, and not tympanitic. The trunk of the body and the limbs were a little augmented in size, and crepitated upon being pressed; the impression of the finger was immediately effaced. The crepitation was so distinct, that, when the nurses were assisting the patient to rise from bed, they thought some of his bones were fractured; and the same noise continued until his death, every time he moved. The dressings were soiled by a reddish-looking serum, which flowed abundantly from the wound.

From this time, the progress of the case was unfavorable: a gangrenous odour issued from the wound, and the man died on the 28th.

*Dissection, six hours after death.* Thermometer indicating two degrees above zero. Emphysema less evident than during life, and only visible in the scrotum and at the upper part of the thigh on the side operated upon. At these parts the skin was tense, and elevated by numerous small phlyctenæ, close together, and containing reddish serum. The flaps of the wound had not adhered to the parts beneath; they were livid, and partially gangrenous. In every point of the cellular tissue where a bistoury was introduced, gas escaped, and, upon the approach of a candle, there was a slight detonation, and it burnt with a brilliant flame of a blueish white colour, whiter at its summit and bluer at the base. From the scrotum and the left thigh the jet of gas was stronger, and lasted longer. The gas was completely inodorous. Muscular system healthy, but the colour of the muscles not so red as usual, and their fibres were separated by well-defined intervals. When cut upon, the same kind of sound was produced as upon the division of muscles that have been slightly frozen. When the muscles were pressed before a light, there was a sparkling and cracking like that which is caused by squeezing out the essential oil from orange-peel before a taper. Upon opening the head, the cerebrum powerfully raised the dura mater; and, wherever this membrane was divided, a hernia cerebri occurred. The brain was light, and, when an incision was made into its substance, a slight noise was heard, similar to that from the section of the muscular tissue; but, upon pressing portions of the cerebral substance before a bougie, neither cracking nor sparks were produced. No gas in the cavities of the heart. In the right auricle and ventricle were two coagula; the largest was in the ventricle, and its size was about that of a filbert. This coagulum was emphysematous; for, upon being brought into contact with a light, and pressed, sparks were emitted and a crackling noise. The blood-vessels were empty. The small transparent veins appeared to contain the same gas. The abdomen was slightly tympanitic, and, upon being punctured, and a light brought near, a clear and blueish smoke was seen, and the puncture was slightly enlarged by the combustion of its edges, which were also blackened. The small intestines contained a very trifling quantity of pus.

It is to be regretted that an accident happened to a vessel in which some of the gas was set aside, for the purpose of ascertaining its precise nature.

This case certainly merits the attention of physiologists, if it were only on account of the presence of the inflammable gas; and those physicians who have thought that spontaneous combustion depended upon inflammable gas being developed in the cellular tissue will here find some support to their theory.

In our Journal for September 1829, p. 280, our readers will find the general results obtained from fifteen cases of spontaneous human combustion; and in our number for June 1831, p. 514, a remarkable case is detailed, which occurred at the Hospital Cochin, of general emphysema, formed by a combustible gas. The 43d and 44th volumes of the Philosophical Transactions contain the description of several cases, and much curious speculation as to the probable causes of spontaneous combustion. A paper upon this subject was also recently published in the Glasgow Medical Journal.—EDITORS.

